# The impact of travel time on colorectal cancer stage at diagnosis in a privately insured population

**DOI:** 10.1186/s12913-019-4004-6

**Published:** 2019-03-18

**Authors:** Mesnad Alyabsi, Mary Charlton, Jane Meza, K. M. Monirul Islam, Amr Soliman, Shinobu Watanabe-Galloway

**Affiliations:** 1 Department of Population Health Research, King Abdullah International Medical Research Center (KAIMRC), King Saud bin Abdulaziz University for Health Sciences, P.O. Box 3660, Riyadh, 11481, 1515 Saudi Arabia; 2 Department of Epidemiology, University of Iowa College of Public Health, 145 N. Riverside Drive, Iowa City, Iowa, 52242 USA; 3 Department of Biostatistics, University of Nebraska Medical Center, College of Public Health, 984375 Nebraska Medical Center, Omaha, NE 68198–4395 USA; 4 Department of Epidemiology, University of Nebraska Medical Center, College of Public Health, 984395 Nebraska Medical Center, Omaha, NE 68198–4395 USA; 5 City University of New York School of Medicine, Community Health and Social Medicine, 160 Convent Avenue, New York, NY 10031 USA

**Keywords:** Access to care, Colorectal cancer, Health services research, Geography, Private insurance

## Abstract

**Background:**

Rural residents are less likely to receive screening for colorectal cancer (CRC) than urban residents. However, the mechanisms underlying this disparity, especially among people aged 50–64 years old with private health insurance, are not well understood. We examined the impact of travel time on stage at CRC diagnosis.

**Methods:**

This retrospective cohort study used data from the Blue Cross and Blue Shield of Nebraska. Members of this private insurance company aged 50–64 years, diagnosed with CRC during the period 2012–2016, and continuously enrolled in the insurance plan for at least 6 months prior to CRC diagnosis, were selected for this study. Using Google Maps, we estimated patients’ travel time from their home ZIP code to the ZIP code of their colonoscopy provider. Using logistic regression, we analyzed the association between stage at CRC diagnosis, travel time, use of preventive services (i.e., check-ups or counseling to prevent or detect illness at an early stage) and patient characteristics.

**Results:**

A total of 307 subjects met the inclusion criteria. People who had not used preventive services 6 months prior to CRC diagnosis had 2.80 (95% CI, 1.00–7.90) times the odds of metastatic CRC compared to those who had used these services. No statistically significant association was found between travel time and metastatic CRC diagnosis (*P* = 0.99; 95% CI, 0.98–1.01).

**Conclusions:**

The fact that 13% of the study population presented with metastatic CRC suggests some noncompliance with preventive services such as screening guidelines. To increase screening uptake and reduce metastatic cases, employers should offer incentives for their employees to make use of preventive services such as CRC screening.

## Background

Colorectal cancer (CRC) is the third most common cancer in the USA, preceded by lung and breast cancers in women, and lung and prostate cancers in men. It is the third leading cause of cancer death in the USA [1, 2]. Unlike breast cancer screening, screening for CRC not only leads to early detection of cancer, but also can prevent cancer from occurring [3, 4]. The large decline in both incidence and mortality from CRC in the USA over the past two decades has been attributed to the increased use of screening, especially colonoscopy [5]. Since the publication of the first guidelines for CRC screening in 1995, the screening rate has steadily increased from 35 to 62.4% in 2015 [6]. In 1998, Congress enacted a Medicare CRC screening benefit (i.e., Medicare is the federal health insurance program for individuals 65 years or older, certain younger individuals with disabilities and individuals with end-stage renal disease), but it was limited to fecal occult blood tests (FOBT) and sigmoidoscopy for symptomatic or high-risk individuals, and average-risk individuals with a positive FOBT who needed follow-up screening [7]. In 2001, the Medicare coverage extended to average-risk individuals nationwide for screening purposes [7, 8]. In 2010, the Affordable Care Act (ACA), or the comprehensive health care reform law enacted in March 2010, required private health insurance plans to cover preventive services recommended by the United States Preventive Services Task Force and graded ‘A’ or ‘B’, including CRC screening, with no out-of-pocket costs for members [9]. By 2011, 54 million privately insured Americans received additional preventive services coverage as required by the ACA, which included colonoscopy screening, mammograms, and Pap smears [10].

Despite its known benefits, CRC screening uptake has remained less than optimal. In 2015, only 65% of Americans eligible for screening were up to date on CRC screening [11], which is well below the 2014 Centers for Disease Control and Prevention’s Colorectal Cancer Program target of 80% [11], and the 70% goal set by Healthy People 2020 [12]. Further, individuals living in rural areas and other medically underserved populations have lower rates of CRC screening uptake. Studies have demonstrated that rural residents are 30% less likely to receive CRC screening than their urban counterparts [13, 14]. Individuals who are underinsured or those with lower socioeconomic status are also less likely to be up to date with CRC screening [15, 16]. While previous research generally suggests lower CRC screening rates among rural residents compared to urban residents, the mechanism to explain this disparity has not been well established. Nevertheless, the following factors are thought to be involved: lower income levels [16, 17], a higher percentage of people who are underinsured or uninsured [15, 18, 19], less awareness and understanding of CRC risks and benefits of CRC screening [20, 21], lack of physician recommendations on CRC screening [18, 22, 23], and distance to a CRC screening facility [24, 25]. For example, a population-based survey study conducted in Nebraska found that rural residents are more likely to perceive screening cost as a barrier, and that CRC cannot be prevented [20]. Unlike urban residents in Nebraska, rural residents are 60% less likely to receive any CRC screening, and 57% less likely to receive a colonoscopy [20].

Furthermore, the rural-urban disparities in access to cancer services is a global phenomenon. Prior international literature assessed the association between rural-urban status and cancer outcome [26]. For instance, Carriere et. Al., 2018 conducted an international systematic review to assess whether cancer survival among rural dwellers is less than their urban counterpart. Despite heterogeneities between studies (e.g., different definitions for rural-urban status and diverse studied geographic regions), rural residents were 5% less likely to survive cancer. Additionally, an Australian systematic review study found less mammography use among rural population which also have contributed to the lower breast cancer survival among women live in rural areas [27].

Many previous CRC and breast cancer screening studies were based on Medicare data, partly because it provides claims data for nearly every US resident over the age of 65 years. It can also provide more objective and reliable evidence for cancer screening use compared to self-reported survey data. However, substantial limitations of Medicare data are the restriction of the population to adults older than 65 years old and the representation of only fee-for-service Medicare beneficiaries. Given the consistently lower use of CRC screening among adults between 50 and 64 years of age [28], it is essential to elucidate factors associated with lower CRC screening in this younger age group. This is especially important given the increased incidence of CRC among individuals younger than 65 years of age [29].

Colonoscopy screening is associated with logistical hurdles such as taking time off work, and having another adult to accompany and transport the patient after the procedure. This is necessary since most patients will undergo conscious sedation and will miss a day of work [30]. Additionally, the unpleasant experience of bowel preparation before the procedure is a common obstacle [20, 23, 31, 32]. These barriers are more likely to be a perceived problem among rural residents since they are more likely to have to travel longer distances to receive a colonoscopy. According to the National Household Travel Survey, 41% of trips taken by rural residents in the US to receive medical or dental services were longer than 30 min in duration, while only 25% of trips taken by urban residents were longer than 30 min [33]. Distance to a screening facility has been shown to be a significant barrier for rural patients [25, 34].

Well-established evidence has suggested a relationship between screening and the prevention and early detection of CRC; therefore, this study was undertaken to assess the impact of travel time on stage at CRC diagnosis among privately insured, rural-dwelling people aged 50–64. We hypothesize that shorter travel time to colonoscopy facilities is associated with earlier stage of diagnosis (i.e., non-metastatic diagnoses) of CRC after accounting for other patient characteristics.

## Methods

### Data sources

This is a retrospective cohort study using data from Blue Cross and Blue Shield of Nebraska (BCBSNE). BCBSNE is the largest private health insurer in Nebraska, serving over 700,000 people [33]. Data comprises claims from inpatient and outpatient facilities, and professional/office services, and include diagnosis and procedural codes, dates of service and the five-digit ZIP code for the provider. The data also contain members’ demographic information including age, gender, and their ZIP code of residence. BCBSNE captures members’ enrollment information including the beginning and end date of coverage. Rural-Urban Community Area Codes (RUCA) data and Google Maps were used to determine rurality and travel time [35, 36].

RUCA uses the U.S. Census Bureau’s Urbanized Area (UA) and Urbanized Cluster (UC) definitions supplemented with information on work commute and population density to characterize all of the census tracts regarding their rural and urban status [36]. The classification assigns metropolitan (i.e., primary commuting flow within an UA), micropolitan (i.e., large rural or primary flow within an UC of 10,000 to 49,999 individuals), small town (i.e., primary flow within an UC of 2500–9999 individuals) and rural commuting areas (i.e., primary flow to a tract outside a UA or UC) with numbers between 1 and 10. These numbers are subdivided into 21 secondary codes based on commuting flows. Although the original RUCA classification was based on census tract, it uses the ZIP code as its geographic unit. The latest RUCA codes are based on the 2010 decennial census and the 2006–2010 American Community Survey.

Furthermore, Google maps offer accurate driving directions between places at no cost. A program was developed (open-source programming language that is available on SAS) to make repeated calls to Google to obtain travel time information for any number of locations [37]. Subsequently, the program was tested using a nationally representative sample that cover 66,000 locations in the fifty states, the District of Columbia and Puerto Rico [35]. The program developer found a high correlation of Google maps with straight-line distance (r^2^ = 0.96) but with superior travel time estimate. While other GIS mapping systems offer greater sophistications and precision, it is at the expense of acquiring and managing specialized GIS software. The additional precision offered by other GIS, doesn’t provide more value to the research question raised on this study (i.e., nonemergency medical care) and to the state of Nebraska [35, 38].

### Study population

**Fig. 1:**
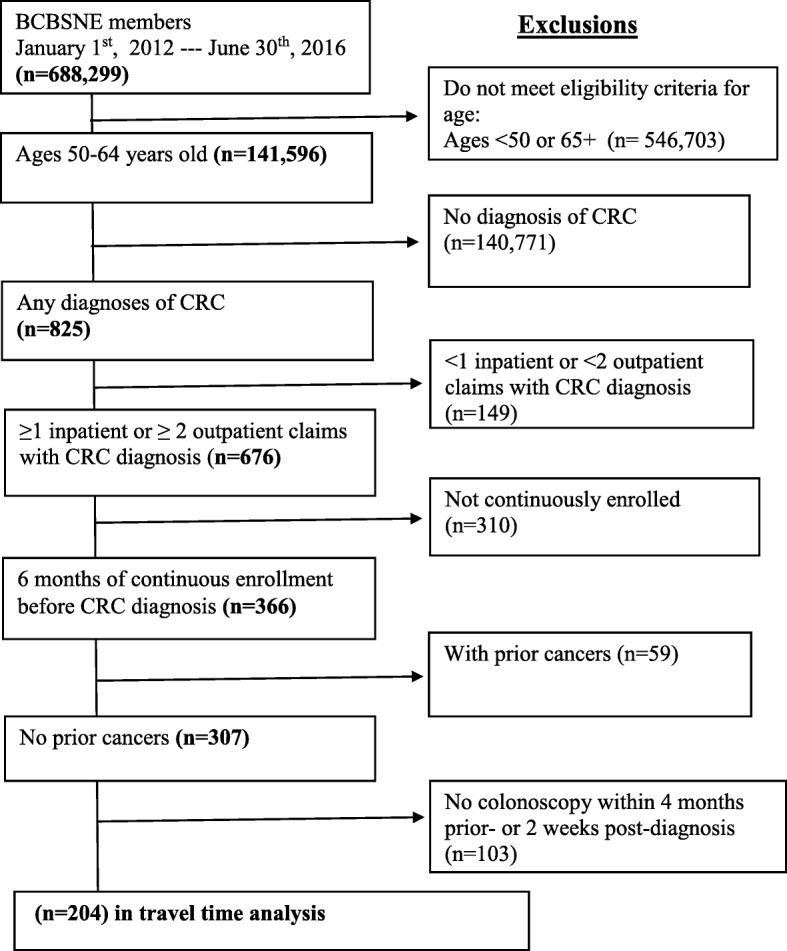
Eligibility criteria for the study population

Individuals included in the study were members of BCBSNE between January 1st, 2012, and June 30th, 2016; aged 50–64 years old with no prior CRC-related claims in the last 3 months; no prior cancers of any kind during the 6 months preceding the index diagnosis [39, 40]: The International Classification of Diseases, Ninth Revision, Clinical Modification (ICD-9-CM) diagnosis codes 140–1529,1550–1958, or 1991–200; ICD-10-CM diagnosis codes C000-C179, C220-C768, or C80-C83; and were enrolled in BCBSNE for at least 6 months prior to the month in which their first diagnosis of CRC was identified. Figure 1 illustrates the eligibility criteria and the number of patients excluded for each criterion. Individuals were considered to be diagnosed with CRC if they had at least one inpatient or two outpatient claims with a primary diagnosis of CRC at two different visits. See the Appendix 1 for the codes used to identify the diagnosis of CRC [41]. We excluded members who were older than 65 years of age because the BCBSNE data likely did not contain claims for all of their Medicare-covered health services. We also excluded members with prior cancers (i.e., cancers at sites other than the colon/rectum) to ensure that the secondary malignant neoplasm originated from CRC [39, 40]. Any members residing outside the state of Nebraska were excluded because the study is confined to the residents of Nebraska.

### Study variables

#### Patient characteristics

The beginning and end dates of coverage and services were extracted from patients’ enrollment files. Patient demographics including age, gender, and five-digit ZIP code of residence were extracted from their membership file. Utilization and clinical variables such as stage at diagnoses and preventive services use were derived from international classification of disease fields, and current procedural terminology fields from the claims file.

A dichotomous yes/no variable was also created to reflect members’ use of preventive services in the 6 months preceding the CRC diagnosis. Preventive services were defined as any health services such as check-ups or counseling to prevent or detect illness at an early stage when treatment is more viable [42]. Codes used to identify preventive services are detailed in the Appendix 2 [43]. In this study, individuals with at least one claim indicating the use of preventive services within 6 months prior to CRC diagnosis were considered to have used preventive services. Initial prevention medicine for a new patient, or periodic prevention medicine for an established patient aged 40–64 years old were examples of the codes used [44].

#### Travel time measurement

A patient’s ‘index’ colonoscopy was designated as the first time a patient had undergone a colonoscopy within 4 months prior to their CRC diagnosis. This colonoscopy was the basis on which both members’ and providers’ ZIP codes were defined to calculate travel time. The provider’s ZIP code was defined as that on the date of the index colonoscopy, and the member’s ZIP code as that for their place of residence when receiving the index colonoscopy.

Travel time was calculated by measuring the time in minutes between the geographic centroid of each member’s ZIP code of residence and the provider’s ZIP code at the time of service. Travel time calculations were made using the Google Maps web page, using the SAS FILENAME URL method in SAS [35]. The method has a high correlation with straight-line distance (r^2^ = 0.96), but with a superior travel time estimate [35]. We measured travel time as a continuous variable, as well as four categories based on quartile distribution. For the rural-urban status definition, we used RUCA codes to assign each member’s residential status based on their residential ZIP code. Subsequently, we used these codes to classify members by rural–urban status using “Categorization C” as suggested by the publisher [45]. This categorization aggregates RUCA codes into urban and rural codes. The urban codes consist of a metropolitan area core, micropolitan or small-town high-commuting areas, or rural areas with a secondary commuting flow of 30 to 49% within an urban area. The rural codes consist of a micropolitan area core with secondary flow of 10–29% to an urban area, small-town, low-commuting areas, or rural areas with commuting to urban cluster areas.

#### Outcome variables

The primary outcome was the stage at CRC diagnosis, classified as metastatic versus non-metastatic CRC. This was based on the initial CRC diagnosis identified in the claims data. The following diagnosis codes were used to identify patients diagnosed with metastatic CRC at the time of initial cancer diagnosis: ICD-9-CM diagnosis codes 196.0, 196.1, 196.3, 196.5, 197.0–197.4, 197.6197.7, 198.0–198.8 or 199.0; and ICD-10-CM diagnosis codes C770, C771, C773, C774, C780.0-C784, C786, C787, C788, C790–C798 or C80. The first date of metastatic diagnosis was assigned as the ‘index’ metastatic diagnosis [39]. Those patients who did not have a metastatic CRC diagnosis at the time of initial cancer diagnosis were considered non-metastatic.

### Data analysis

Age, gender, rural-urban status, colonoscopy use within 4 months prior to CRC diagnosis, use of preventive services, and travel time were compared between metastatic and non-metastatic groups using Wilcoxon’s rank-sum test for continuous variables, and the Chi-square (X^2^) test for categorical variables. Wald tests were used to assess the significance of predictors. We used the fractional polynomial method to examine any non-linear relationships between the log odds of metastatic diagnosis and continuous variables. We inspected the curves of the predictors against the dichotomous response and used the likelihood ratio test for improvement in fit against the assumed linear relationship. Lastly, we conducted a multivariate analysis to assess the relationship between travel time and metastatic CRC diagnosis, adjusting for gender, rural-urban status, and use of preventive services. All tests were two-tailed and with α = 0.05. We used SAS statistical software version 9.4 (SAS Institute Inc. Cary, NC) to conduct all analyses.

## Results

The application of the inclusion and exclusion criteria resulted in a cohort of 307 patients (Fig. 1). Of the 307 members who met our eligibility criteria, we were able to identify that 66% (*n* = 204) had undergone a colonoscopy within the 4 months prior to CRC diagnosis; 13% (*n* = 27) of these presented with metastatic CRC. There were no differences in the measured characteristics between patients who used colonoscopy within the 4 months prior to CRC diagnosis and those who did not. Table 1 shows the characteristics of BCBSNE members diagnosed with CRC who made a claim for colonoscopy within the 4 months prior to CRC diagnosis. There were no significant differences between metastatic and non-metastatic cases. Most cases were diagnosed during the years 2013 and 2014 in both metastatic and non-metastatic groups. While the average number of months of enrollment before CRC diagnosis were similar between metastatic and non-metastatic cases (13.0 versus 13.53), the median number of months of enrollment was slightly higher among the metastatic group (26.0 versus 22.0).

**Table 1:** Characteristics of BCBSNE members diagnosed with CRC who made a colonoscopy claim within 4 months prior to their CRC diagnosis (*n* = 204)

Characteristics	Total (204)		Metastatic (27)		Non-metastatic (177)		
	N	%	N	%	N	%	*P*
Age							
Mean (SD)	57.50	4.0	57.56	4.56	57.48	4.15	0.91
Median (SD)	58.0	7.0	57.0	8.0	58.0	7.0	
50–54	56	27.0	7	26.0	49	28.0	
55–60	92	45.0	12	44.0	80	45.0	
≥ 61	56	28.0	8	30.0	48	27.0	
Gender							
Male	112	54.90	17	63.0	95	53.67	0.37
Female	92	45.10	10	37.0	82	46.33	
Member location							
Rural	110	53.92	11	41.0	99	56.0	0.14
Urban	94	46.08	16	59.0	78	44.0	
Travel time (min)							
Mean (SD)	33.58	40.0	34.85	51.53	33.38	38.12	0.74
Median (SD)	19.0	27.0	18.0	17.0	19.0	28.0	
Q1	11	–	13	–	11	–	
Q2	19	–	18	–	19	–	
Q3	38	–	30	–	39	–	
Q4	227	–	223	–	227	–	
Travel distance, (miles)							
Mean (SD)	29.0	43.0	30.0	58.0	29.0	40.50	0.74
Median (SD)	13.0	26.0	13.0	19.0	13.0	28.50	
Q1	5.0	–	5.0	–	4.50	–	
Q2	13.0	–	13.0	–	13.0	–	
Q3	31.0	–	24.0	–	33.0	–	
Q4	251.0	–	251.0	–	234.0	–	
PCP access							
PCP/10,000 mean	11.03	8.10	12.33	12.08	10.82	7.31	0.95
PCP/10,000 median	10.08	8.51	7.82	13.58	10.24	8.19	
Year of CRC diagnosis							
2012	33	16.0	7	26.0	26	15.0	
2013	50	25.0	4	15.0	46	26.0	
2014	62	30.0	11	41.0	51	29.0	
2015	39	19.0	5	18.0	34	19.0	
2016	20	10.0	0	0	20	11.0	
Months of enrollment before CRC diagnosis							
Mean (SD)	26	13.50	25	13.0	26	13.53	
Median (IQR)	26	22.0	28	26.0	25	22.0	

Table 2 shows our analyses of the association between travel time and metastatic CRC among members who had undergone colonoscopy within 4 months prior to their diagnosis (*n* = 204). Mean and median travel times were very similar between the metastatic (mean = 34.85 min) and non-metastatic (mean = 33.38 min) groups. Among these members, 25% traveled a distance of a maximum of 6 miles, 50% traveled a distance of a maximum of 15 miles, and 75% traveled a distance of a maximum of 31 miles. While the median distance traveled by rural patients was 26 miles, urban patients traveled a median of 8 miles to get to a colonoscopy facility. For those who did not have claims for preventive services, the odds of being diagnosed with metastatic CRC was 2.80 (95% CI: 1.00–7.90) times greater than those who had claims for preventive services prior to CRC diagnosis.

**Table 2:** Logistic regressions of CRC patients who made a colonoscopy claim within 4 months prior to their CRC diagnosis (*n* = 204)

Characteristics	Univariate Model		Multivariate Model
	Odds ratio (95% CI)	*P*	Odds ratio (95% CI)
Age	1.004 (0.91–1.01)	0.91	
Gender			
Male	1.0		1.0
Female	0.68 (0.29–1.57)	0.37	0.76 (0.32–1.80)
Member location			
Rural	1.0		1.0
Urban	1.84 (0.82–4.20)	0.14	2.14 (0.87–5.30)
Travel time (min)			
Mean	1.001 (0.99–1.01)	0.74	0.99 (0.98–1.01)
Preventive services			
Yes	1.0		1.0
No	2.81 (1.02–7.77)	0.04	2.80 (1.00–7.90)

The association between travel time and metastatic CRC diagnosis is adjusted for gender, rural–urban status, and use of preventive services

## Discussion

The motivation to conduct this study was based on the notion that, unlike the Medicare population, people aged 50–64 years old more often have barriers related to work schedules, and are therefore less inclined to travel to a colonoscopy facility to be screened, leading to more frequent presentation with metastatic CRC [46–48]. Although our focus was primarily on the impact of rurality and travel times, we also examined the roles of demographic factors and use of preventive services; the characteristics of people in rural populations are different from those of the urban population, and access to services is of concern [20, 25, 49, 50].

We found no significant association between rural residency and late-stage at CRC diagnosis, which is comparable to results from recent studies that used cancer registry data from Iowa, Nebraska, and Georgia [50–52], with exceptions from a study that used 1998–2002 Illinois cancer registry data [53]. Also, the current study did not find a significant association between travel time and late-stage diagnosis. Again, the findings from this study generally confirm the results from the studies that used cancer registry data [51, 54]. Also, survey data generally indicate a lower adherence rate of CRC screening among rural residents compared to urban residents [13, 55].

Another explanation for the discrepancy between the survey studies and our results could be attributed to the differences between registry and claims data versus survey data. Survey data include insured, uninsured or underinsured individuals, while our sample consists of only insured individuals. Survey data can also suffer from potential biases (e.g., recall bias, social desirability bias), while registry data, and claims-based data, are more valid because of the accuracy required when reporting for a registry, and because claim’s reimbursement is dependent on patients’ health encounters. Additionally, registry data covers the entire state of Nebraska. While our sample is from BCBSNE, which is the largest private health insurance in Nebraska [56], the sample does not represent the entire privately insured population in Nebraska, nor the uninsured population. Another likely explanation is that there are widely varying degrees of rurality in different states. Rural Nebraska is very different from rural Alaska, or rural Kentucky, for example, where the differences between urban and rural populations in terms of use of health services may be much greater.

Our results indicate the key role of preventive services in the prevention or early detection of CRC. We found that patients who did not use preventive services within the 6 months prior to their CRC diagnosis were two times more likely to be diagnosed as having metastatic CRC compared to those who had availed of such services. This result likely reflects the notion that cancer screening communication between the patient and the provider may occur during an annual checkup or other routine care settings [57–59]. Not surprisingly, it also likely demonstrates that preventive visits are the main referral source for screening colonoscopies, whereas acute visits for CRC-related symptoms are the main referral source for diagnostic colonoscopies. Accordingly, non-distance barriers that can prevent or delay the receipt of preventive services should be alleviated. For instance, to encourage visits to a primary care physician, insurers/employers should consider removing cost-sharing during the initial CRC screening visits. An example of a potential cost that might incur during the initial visit is polyp removal [60, 61]. Co-insurance should also be removed from those undergoing follow-up colonoscopies after positive stool-based screening tests.

Compared to the older population (aged ≥65 years), CRC in the younger population appears to be a more aggressive disease [62–67]. It is more prevalent in the distal colon or rectum, tends to be poorly differentiated, more likely to be of mucinous and signet ring feature, and is typically diagnosed at later stages. These characteristics have been associated with a fast-growing CRC [68–70]. In addition to differences in molecular biology, the more aggressive nature among younger patients could be a result of lack of screening, non-compliance with screening guidelines, or failure to recognize and assess colonic symptoms among younger subjects [29, 69, 71, 72]. Non-compliance with colonoscopy screening has been linked to logistical barriers such as scheduling the colonoscopy procedure, or taking time off work to undergo colonoscopy, which is more common among the working-age population [73, 74].

Some limitations should be noted when interpreting the findings. First, we excluded 33.6% of the study sample for the travel time analysis because we were not able to identify whether these individuals had made colonoscopy claims within the 4 months prior to their CRC diagnosis. It is possible that some of these patients were diagnosed at the time of surgery because of an obstructed colon. Another possible explanation was that some of these cases could have been initially diagnosed with CRC prior to the study period, and then later presented with metastatic CRC that was diagnosed via radiologic imaging. Nonetheless, excluded cases were not significantly different from those included in all measured characteristics in this study. Second, our population is limited to individuals with private insurance from BCBSNE who live in the state of Nebraska, and our results may therefore not generalize to populations located in different states or with different types of insurance. Nonetheless, Nebraskans represent the US population relatively well in terms of sociodemographic characteristics except Nebraska has a higher proportion of whites than the U.S. average (79.0% vs. 60.7%) [75]. Third, we were unable to determine whether members bypassed the closest colonoscopy facility and voluntarily traveled longer distances to undergo screening at other facilities because BCBSNE data does not include all colonoscopy facilities in the state of Nebraska. Fourth, previous studies show that SES is proportionally associated with access to health services use [76]. In the current study, we were unable to control for the effect of SES. Nonetheless, the studied population is comprised of privately-insured population who we assumed is a homogenous population. We considered insurance status a proxy to SES because it has been found to be important in determining health outcomes in terms of access to health care [76]. According to US census data, 93.4% of Nebraskans have obtained a high school diploma and 59% of the households with median earnings of ≥ $45,000 [77]. Fifth, findings should be interpreted with cautions since the 6 months period for identifying preventive services use might not entirely capture the health behavior of an individual. For instance, one might use more health services only during or after an acute illness and thus, will be less likely to be captured within the window of 6 months. Ideally, we should examine the preventive services use within longer period of CRC diagnosis [44, 78]. Finally, it is possible that the current study is underpowered to find association between travel-time and stage at diagnosis.

## Conclusions

This study did not find an association between the travel time taken for patients to reach colonoscopy facilities and the metastatic stage of their cancer at diagnosis. The fact that 13% of this privately insured working-age population presented with metastatic CRC suggests some non-compliance with screening guidelines. It is also possible that in this young cohort, some cases presented with an aggressive and fast-growing disease, with the potential development of metastatic CRC between screening colonoscopies. Nevertheless, it is possible that this working-age population faces logistic barriers that prevent them from taking time off work to undergo screening. To increase screening use and reduce cases of metastatic CRC, employers should offer incentives (e.g., removing cost-sharing during initial visits, or for follow-up colonoscopy among individuals with FOBT positive test) for their employees to undergo preventive services such as CRC screening.

## Appendix 1

**Table 3:** ICD codes used in identifying the diagnosis of CRC

ICD code-9 and 10	Description
153.0/C183	Malignant neoplasm of hepatic flexure
153.1/C184	Malignant neoplasm of transverse colon
153.2/C186	Malignant neoplasm of descending colon
153.3/C187	Malignant neoplasm of sigmoid colon
153.4/C180	Malignant neoplasm of cecum
153.6/C182	Malignant neoplasm of ascending colon
153.7/C185	Malignant neoplasm of splenic flexure
153.8/C188	Malignant neoplasm of other specified sites of large intestine
153.9/C189	Malignant neoplasm of colon, unspecified site
154.0/C19	Malignant neoplasm of rectosigmoid junction
154.1/C20	Malignant neoplasm of rectum
154.8/C218	Malignant neoplasm of other sites of rectum, rectosigmoid junction, and anus

## Appendix 2

**Table 4:** ICD and CPT codes used in identifying preventive services use

ICD code-9 and 10	Description	CPT code	Description
V700	General medical examination, routine medical examination at health care facility	99,386	initial preventive medicine new patient 40-64 yrs
V708	General med. Exam., other specified general medical examinations	99,396	periodic preventive med established patient 40-64 yrs
V709	General med. Exam., unspecified general medical examination		
Z0000	Encounter for general adult medical examination		
Z008	Encounter for other general exam		
V7231	Routine gynecological examination		
V7232	Pap smear confirmation		
Z0141	Encounter for routine gynecological examination		
Z01411	Encounter for routine gynecological examination with abnormal findings		
Z01419	Encounter for routine gynecological examination, no abnormal findings		
Z0142	Confirms pap smear test		

## Abbreviations

ACA: Affordable Care Act
BCBSNE: Blue Cross Blue Shield of Nebraska
CRC: Colorectal Cancer
FOBT: Fecal Occult Blood Test
ICD: International Classification of Diseases
RUCA: Rural Urban Commuting Area
